# Quantitative study of medicinal plants used by the communities residing in Koh-e-Safaid Range, northern Pakistani-Afghan borders

**DOI:** 10.1186/s13002-018-0229-4

**Published:** 2018-04-25

**Authors:** Wahid Hussain, Lal Badshah, Manzoor Ullah, Maroof Ali, Asghar Ali, Farrukh Hussain

**Affiliations:** 1 Department of Botany, University of Peshawar, Peshawar, 25000 Pakistan; 2 Department of Botany, University of Science and Technology, Bannu, Pakistan; 3 Department of Plant Science, Quaid-i-Azam University, Islamabad, Pakistan; 4 Dr. Khan Shaheed Govt. Degree College Kabal, Swat, Pakistan; 5 Institute of Biological Sciences, Sarhad University of Science and Information Technology, Peshawar, Pakistan

**Keywords:** Quantitative study, Medicinal plants, Traditional knowledge, Koh-e-Safaid Range

## Abstract

**Background:**

The residents of remote areas mostly depend on folk knowledge of medicinal plants to cure different ailments. The present study was carried out to document and analyze traditional use regarding the medicinal plants among communities residing in Koh-e-Safaid Range northern Pakistani-Afghan border.

**Methods:**

A purposive sampling method was used for the selection of informants, and information regarding the ethnomedicinal use of plants was collected through semi-structured interviews. The collected data was analyzed through quantitative indices viz. relative frequency citation, use value, and family use value. The conservation status of medicinal plants was enumerated with the help of International Union for Conservation of Nature Red List Categories and Criteria (2001). Plant samples were deposited at the Herbarium of Botany Department, University of Peshawar for future reference.

**Results:**

One hundred eight informants including 72 male and 36 female were interviewed. The informants provided information about 92 plants species used in the treatment of 53 ailments. The informant reported maximum number of species used for the treatment of diabetes (16 species), followed by carminatives (12 species), laxatives (11 species), antiseptics (11 species), for cough (10 species), to treat hepatitis (9 species), for curing diarrhea (7 species), and to cure ulcers (7 species), etc. Decoction (37 species, i.e., 40%) was the common method of recipe preparation. Most familiar medicinal plants were *Withania coagulans*, *Caralluma tuberculata*, and *Artemisia absinthium* with relative frequency (0.96), (0.90), and (0.86), respectively. The relative importance of *Withania coagulans* was highest (1.63) followed by *Artemisia absinthium* (1.34), *Caralluma tuberculata* (1.20), *Cassia fistula* (1.10), *Thymus linearis* (1.06), etc. This study allows identification of novel uses of plants. *Abies pindrow*, *Artemisia scoparia*, *Nannorrhops ritchiana*, *Salvia reflexa*, and *Vincetoxicum cardiostephanum* have not been reported previously for their medicinal importance. The study also highlights many medicinal plants used to treat chronic metabolic conditions in patients with diabetes.

**Conclusions:**

The folk knowledge of medicinal plants species of Koh-e-Safaid Range was unexplored. We, for the first time, conducted this quantitative study in the area to document medicinal plants uses, to preserve traditional knowledge, and also to motivate the local residents against the vanishing wealth of traditional knowledge of medicinal flora. The vast use of medicinal plants reported shows the significance of traditional herbal preparations among tribal people of the area for their health care. Knowledge about the medicinal use of plants is rapidly disappearing in the area as a new generation is unwilling to take interest in medicinal plant use, and the knowledgeable persons keep their knowledge a secret. Thus, the indigenous use of plants needs conservational strategies and further investigation for better utilization of natural resources.

**Electronic supplementary material:**

The online version of this article (10.1186/s13002-018-0229-4) contains supplementary material, which is available to authorized users.

## Background

The residents of remote areas mostly depend on folk knowledge of medicinal plants to cure different ailments. Plants not only provide food, shelter, fodder, drugs, timber, and fuel wood, but also provide different other services such as regulating different air gases, water recycling, and control of different soil erosion. Hence, phytodiversity is required to fulfill several human daily livelihood needs. Millions of people in developing countries commonly derive their income from different wild plant products [1]. Ethnomedicinal plants have been extensively applied in traditional medicine systems to treat various ailments [2]. This relationship goes back to the Neanderthal man who used plants as a healing agent. In spite of their ancient nature, international community has recognized that many indigenous communities depend on biological resources including medicinal plants [3]. About 80% of the populations in developing countries rely on medicinal plants to treat diseases, maintaining and improving the lives of their generation [4, 5]. The people, in most parts of the world particularly in rural areas, rely on traditional medicinal plants’ remedies due to easy availability, cultural acceptability, and poor economic conditions. Out of the total 422,000 known angiosperms, more than 50,000 are used for medicinal purposes [6]. Some 75% of the herbal drugs have been developed through research on traditional medicinal plants, and 25% of prescribed drugs belong to higher plants [7]. Traditional knowledge has a long historical cultural heritage and rich natural resources that have accumulated in the indigenous communities through oral and discipleship practices [8]. Traditional indigenous knowledge is important in the formulation of herbal remedies and isolates bioactive constituents which are a precursor for semisynthetic drugs. It is the most successful criterion for the development of novelties in drugs [9–11]. Traditional knowledge can also contribute to conserve and sustain the use of biological diversity. However, traditional knowledge, especially herbal health care system, has declined in remote communities and in younger generations as a result of a shift in attitude and ongoing socio-economic changes [12]. The human communities are facing health and socio-economic problems due to changing environmental conditions and socio-economic status [13]. The tribal people have rich unwritten traditional medicinal knowledge. It rests with elders and transfers to younger orally. With rapid economic development and oral transmitted nature of traditional knowledge, there is an urgent need to systematically document traditional medicinal knowledge from these communities confined in rural and tribal areas of the world including Pakistan. The Koh-e-Safaid Range is one of the remote tribal areas of Pakistan having unique and century-old ethnic characteristics. A single hospital with limited insufficient health facilities is out of reach for most inhabitants. Nature has gifted the area with rich diversity of medicinal plants. The current advancement in the use of synthetic medicines has severely affected the indigenous health care system through the use of medicinal traditional practices in the area. The young generation has lost interest in using medicinal plants, and they are reluctant to practice traditional health care system that is one of the causes of the decline in traditional knowledge system. Quantitative approaches can explain and analyze the variables quantitatively. In such approach, authentic information can be used for conservation and development of existing resources. Therefore, the present research was conducted in the area to document medicinal uses of local plants with their relative importance, to record information for future investigation and discovery of novelty in drug use, and to educate the locals about the declining wealth of traditional and medicinal flora from the area.

## Methods

### Ethnographic and socio-economic background of the study area

**Fig. 1:**
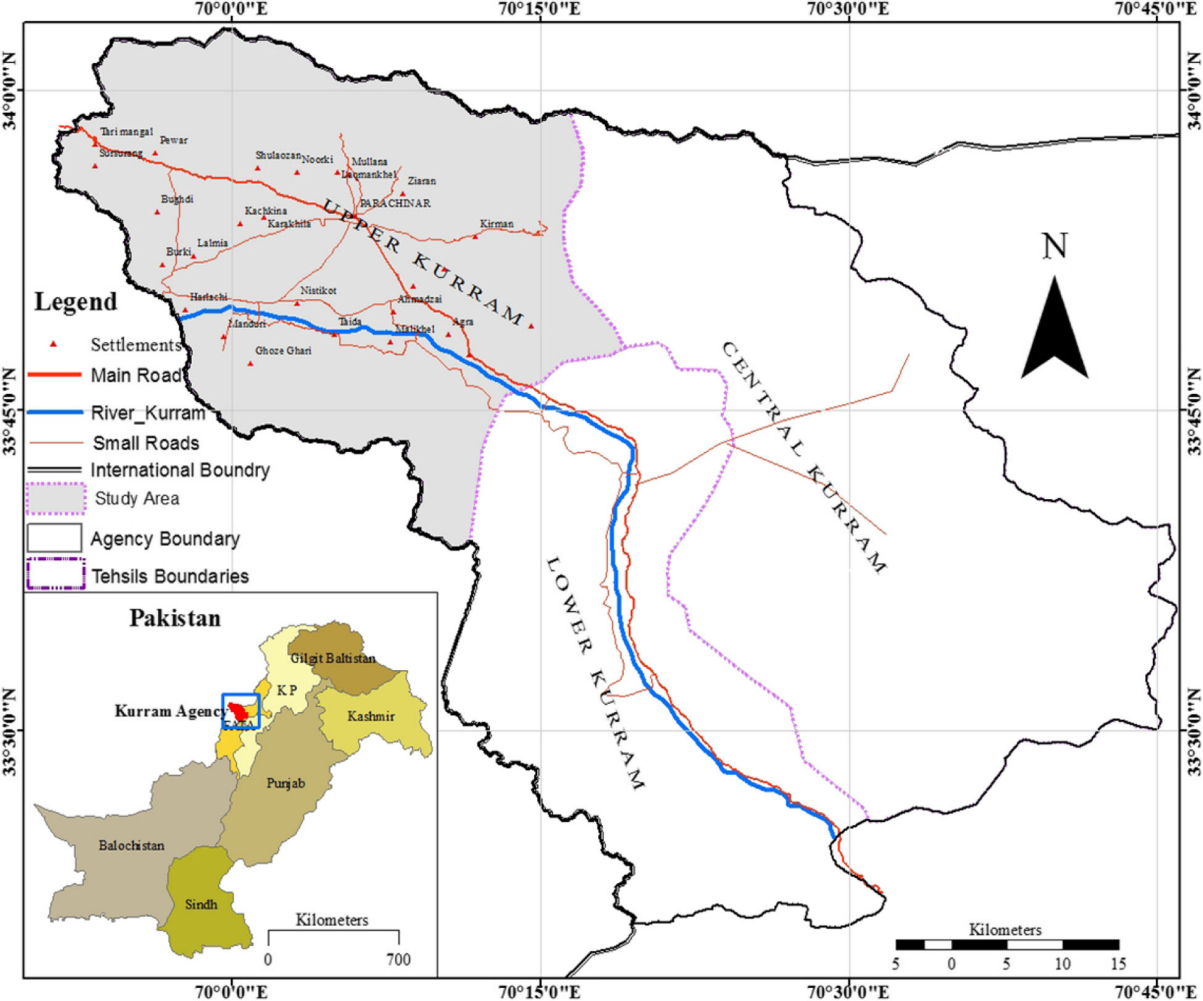
Map of the study area and area location in Pakistan

Koh-e-Safaid Range is a tribal territory banding Pakistan with Afghanistan in Kurram Agency. It lies between 33° 20′ to 34° 10′ N latitudes and 69° 50′ to 70° 50′ E longitudes (Fig. 1).This area is federally administered by the Government of Pakistan. The Agency is surrounded on the east by Orakzai and Khyber agencies, in the southeast by Hangu district, and in the south by North Waziristan Agency and Nangarhar and Pukthia of Afghanistan lies on its west. The highest range of Koh-e-Safaid is Sikaram peak with, 4728 m height. The Agency is well-populated with many small fortified villages receiving irrigation water from Kurram River that flows through it. The weather of the Agency is mostly pleasant in summer; however, in winters, freezing temperature is experienced, and sometimes falls to − 10 °C. The weather charts website “Climate-Charts” ranked it as the fourth coldest location in Pakistan. Autumn and winter are usually dry seasons while summer and spring receive much of the precipitation. The total population of the Agency according to the 2017 censuses report is 253,478. Turi, Bangash, Sayed, Maqbal, Mangel, Khushi, Hazara, Kharote, and Jaji are the major tribes in the research area. The joint family system is practiced in the area. Most of the marriages are held within the tribe; however, there is no ban on the marriages outside the tribe. Marriage functions are communal whereby all relatives, friends, and village people participate with songs, music, and dances male and female separately. The death and funeral ceremonies are jointly attended by the friends and relatives. The people of the area follow Jirga to resolve their social and administrative problems. This is one of the most active and strong social institutions in the area. Economically, most people in the area are poor and earning their livelihoods by menial jobs. The professional includes farmers, pastoralists, shopkeepers, horticulturists, local health healers, wood sellers, and government servants. In the adjoining areas of the city, pastorals keep domestic animals and are considered a better source of income.

### Sampling method

The study was conducted through purposive sampling by informants’ selection method. The selection of informants was primary based on the ethnomedicinal plants and their willingness to share the information. The selection criteria include people who prescribe recipes for treatment; people involved in buying, collection, or cultivation of plants; elder members of above 60 years age; and young literate members. The participants were traditional healers, plant collectors, farmers, traders, and selected knowledgeable elders above 60 years age and young ones. The interviews were conducted in local Pashto language in the local dialect. The informants were involved in the gathering of data with a consent of village tribe chieftains called Maliks.

### Data collection

Semi-structured open-ended interviews were conducted for the collection of ethnomedicinal information from April 2015 to August 2017. Informants from 19 localities were interviewed including Sultan, Malikhail, Daal, Mali kali, Alam Sher, Kirman, Zeran, Malana, Luqman Khail, Shalozan, Pewar, Teri Mangal, Bughdi, Burki, Kharlachi, Shingak, Nastikot, Karakhila, and Parachinar city (Fig. 1). The objectives of this study were thoroughly explained to all the informants before the interview [14]. Data about medicinal plants and informants including local names of plants, preparation of recipes, storage of plant parts, informant age, occupation, and education were collected during face-to-face interviews. A questionnaire was set with the following information: informant bio-data, medicinal plant use, plant parts used and modes of preparation, and administration of the remedies. Plants were confirmed through repeated group discussion with informants [15, 16]. For the identification of plants, informants were requested for transect walks in the field to locate the cited plant for confirmation.

### Collection and identification of medicinal plants

**Fig. 2:**
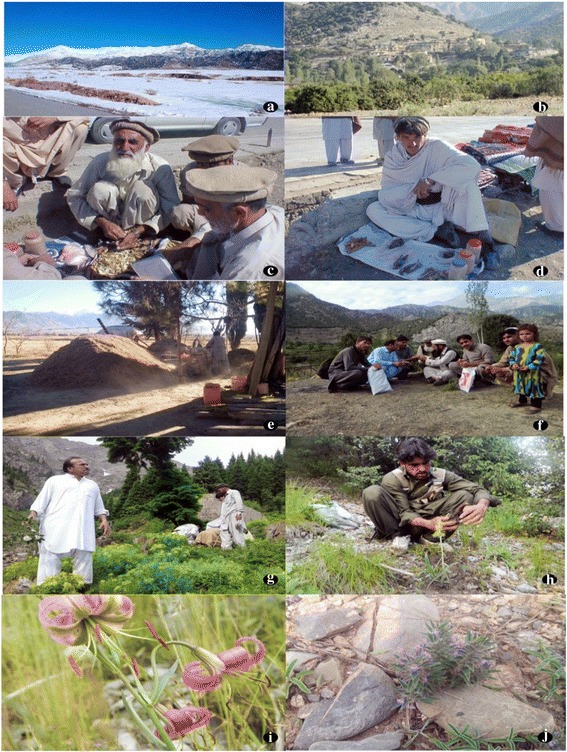
Landscape of Kurram Valley (**a** winter, **b** summer). **c**, **d** Traditional healers selling herbal drugs on footpath. **e** Trader crushing *Artemisia absinthium* for marketing. **f** Principal author in the field during data collection. **g**, **h** Plant collectors in subalpine zone. **i** *Lilium polyphyllum* rare species distributed in subalpine zone. **j** *Ziziphora tenuior* endangered species of subtropical zone

The medicinal plants used in traditional treatment of ailments in the study area were collected with the help local knowledgeable persons, traditional healers, and botanists. The plants were pressed, dried, and mounted on herbarium sheet. The field identification was confirmed by a taxonomist in the Herbarium Department of Botany, University of Peshawar. The voucher specimens of all species were numbered and deposited in the Herbarium of Peshawar University (Fig. 2).

### Data analysis

The information about ethnomedicinal uses of plants and informants included in questionnaires such as botanical name, local name, family name, parts used, mode of preparation, use reports, frequency of citation, relative importance, and voucher number were tabulated for all reported plant species. Informants’ use reports for various ailments and frequency of citation were calculated for each species. The relative importance of species was calculated according to use-value formula (UV = UVi/Ni) [17], where “UVi” is the number of citations for species across all informants and “Ni” the number of informants. The citation probability of each medicinal plant across all informants was equal to avoid researchers’ biasness. Family use value was calculated using the formula FUV = UVs/Ns, where “UVs” represent the sum of use values of species falling within family, and Ns represents the number of species reported for the family. The conservation status of wild medicinal plants species was enumerated by applying International Union for Conservation of Nature (IUCN) criterion (2001) [18].

## Results

### Informants’ knowledge about medicinal plants and their demography

A total of 108 including 72 male and 36 female informants were interviewed from 19 locations. The three groups of male respondents were falling in the age groups of 21 to 40, 41 to 60, and 61 to 80 years having the numbers of 19, 19, and 34, respectively. Among the female respondents, 10 aged 21 to 40, 14 aged 41 to 60, and 12 aged 61 to 80 years. Among the informants, 15 males were illiterate, 34 were matriculate, 13 were intermediate, and 10 were graduates. Among the females, 19 were illiterate, 16 were matriculate, and only 1 was graduate (Table 1). Informants were shepherd, healers, plant collectors, gardeners, and farmers. Twenty-eight informants of above 60 years age, living a retired life, were also interviewed. It was found that males were more knowledgeable than females. Furthermore, health healers were more knowledgeable.

**Table 1:** Demographic detail of informants residing in Koh-e-Safaid Range, upper Kurram

Variables	Categories	Number of informants in each category	Percentage	Sum of reports
Sex ratio	Women	36	33.33	694
	Man	72	66.66	3162
Age groups	Between 20 and 40 years	29	26.85	120
	Between 41and 60 years	33	30.55	660
	Above 60 years	46	42.59	3076
Educational level	Illiterate	38	35.18	914
	Matric	46	42.59	1868
	Intermediate	13	12.03	592
	Graduate	11	10.18	463
Social livelihoods	Farmer	12	11.11	41
	Shepherd	18	16.66	104
	Plant collector	19	17.59	91
	Elder (non-professional)	28	25.92	2052
	Healer	09	8.33	904
	Gardeners	13	12.03	238
	Shopkeeper	04	3.70	15
	Trader	05	4.62	35
Life type	Town area	19	17.59	–
	Remote area	89	82.40	–

### Diversity of medicinal plants

A total of 92 medicinal species including 91vascular plant species belonging to 50 families and 1 mushroom *Morchella* of Ascomycetes of family Morchellaceae were reported (Table 2)*.* Asteraceae had eight species followed by seven species of Lamiaceae and Rosaceae. Three species were contributed by each of Moraceae, Asclepiadaceae, Polygonaceae, Brassicaceae, Solanaceae, Cucurbitaceae, and Liliaceae. Of the remaining eight families, namely, Poaceae, Pinaceae, Zingiberaceae, Chenopodiaceae, Plantaginaceae, Apiaceae, Fabaceae, and Zygophyllaceae, each one contributed two species [19, 20]. Asteraceae, Lamiaceae, and Rosaceae were also reported with a high number of plants used for medicinal purposes. The reported plants were collected both from the wild (86.9%) and cultivated (13.1%) sources. However, greater percentage of medicinal plants from wild sources indicated higher species’ diversity in the study area. The 62 herbs species, 16 tree, 12 shrubs, and 2 undershrubs species were used in medicinal preparation for remedies.

**Table 2:** Enumeration of medicinal plants species in Koh-e-Safaid Range, Kurram, Pakistan

Botanical name/local name/voucher number	Family	Availability	Habit	Part(s) used	Formulation of remedies	ROA	Medicinal use	RFC	Use reports
*Abies pindrow* Royle ex D. Don/Bejoor/B. Huss.55.UOP	Pinaceae	W	Tr	Seeds	Dec	O	Antidiabetic	0.05	5
*Adiantum capillus-veneris* L./Lailazalfi/ B.Huss.56.UOP	Adiantaceae	W	H	Leaves	J	T	Skin problems	0.18	19
*Allium cepa* L./Pyaz/ B. Huss.57.UOP	Amaryllidaceae	C	H	Bulb	Ro	T	Anti-inflammatory	0.84	37
							Antiseptic		2
							Spine removal		52
*Allium sativum* L./Woga/B.Huss.58.UOP	Amaryllidaceae	C	H	Bulbils	Ro/Dir or Veg	O, T	Antiseptic	0.26	5
							Blood pressure		23
*Aloe vera* (L.) Burm. f./Zargoya/B.Huss.59.UOP	Asphodelaceae	C	H	Leaves	Dir	T	Wound healing	0.22	24
*Amaranthus viridis* L./Ranzaka/B.Huss.60.UOP	Amaranthaceae	W	H	Leaves	Veg	O	Laxative	0.34	31
							Blood purifier		6
*Artemisia absinthium* L./Mastyara/ B.Huss.61.UOP	Asteraceae	W	H	Leaves	Dec	O	Antidiabetic	1.34	13
							Antimalarial		93
							Antipyretic		32
							Blood pressure		5
							Urologic problems		2
*Artemisia scoparia* Waldst. & Kit/Doorang/B.Huss.62.UOP	Asteraceae	W	H	Root	Dec	O	Anticancer	0.69	22
							Antidiabetic		41
							Hepatitis		11
*Asparagus adscendens* Roxb./Asparages/B.Huss.63.UOP	Asparagaceae	W	H	Aerial parts	Dec	O	Tonic	0.06	7
*Berberis lycium* Royle/Sur Azghey/B.Huss.64.UOP	Berberidaceae	W	S	Bark	Dec	O	Antiseptic	0.70	14
							Antiulcer genic		8
							Chest problems		37
							Cough		17
*Bergenia ciliata* (Haw)/Qamar Gul/B.Huss.65.UOP	Saxifragaceae	W	H	Aerial parts	Pow	O	Stomach pain	0.18	13
							Joint pain		6
*Calotropis procera* (Ait.) Ait. f., Hort./Sahrashodeky/B.Huss.66.UOP	Asclepiadaceae	W	S	Leaves, latex	Dir	T	Antiseptic	0.76	17
							Wound healing		65
*Cannabis sativa* L./Bangooboti/B.Huss.67.UOP	Asclepiadaceae	C	H	Flowers, leaves	Inf	O	Sedative	0.78	32
							Refrigerant		52
*Caralluma tuberculata* N. E. Brown/Pamenny B.Huss.68.UOP	Asclepiadaceae	W	H	Aerial parts	Dir, Veg	O	Antidiabetic	1.20	97
							Anticancer		3
							Blood purifier		5
							Stomachic		25
*Cassia fistula* L/Toorlargy/B.Huss.69.UOP	Caesalpiniaceae	C	Tr	Fruit	Inf	O	Stomach pain	1.10	34
							Carminative		58
							Colic pain		27
*Chenopodium album* L/Sarmy/B.Huss.70.UOP	Chenopodiaceae	W	H	Leaves	Veg	O	Laxative	0.10	11
*Chenopodium ambrosioides* L/Boi Sarmy/B.Huss.71.UOP	Chenopodiaceae	W	H	Leaves	Dec	O	Anthelmintic	0.08	9
*Cichorium intybus* L/Shin gulay/B.Huss.72.UOP	Asteraceae	W	H	Whole plant	Dec, Veg	O	Antipyretic	0.85	11
							Antidiabetic		17
							Antimalarial		26
							Hepatitis		38
*Citrullus colocynthis* L/Perpendu/B.Huss.73.UOP	Cucurbitaceae	W	H	Fruit	Dec	O, T	Antidiabetic	0.23	23
							Carminative		11
							Refrigerant		14
*Coriander sativum* L/Danya/B.Huss.74.UOP	Apiaceae	C	H	Aerial parts	Dir, Veg	O	Carminative	0.34	35
							Hypolipidemic		16
*Cotoneaster microphyllus* Wall. Ex Lindl/Mamany/B.Huss.75.UOP	Rosaceae	W	S	Fruit, root	Dir, Dec	O	Carminative	0.32	8
							Hepatitis		35
*Crataegus oxycantha* L/Ghunza/B.Huss.76.UOP	Rosaceae	W	Tr	Fruit	Dir	O	Blood pressure	0.14	14
							Dyspnea		9
*Cucurbita maxima* Duch. ex Lam/Kado/B.Huss.77.UOP	Cucurbitaceae	C	H	Fruit	Veg	O	Laxative	0.10	10
							Colic pain		5
*Curcuma longa* L/Korkaman, Hildi/B.Huss.78.UOP	Zingiberaceae	C	H	Rhizome	Dec	O	Wound healing	0.24	26
*Daphne mucronata* Royle/Laghoony/B.Huss.79.UOP	Thymelaeaceae	W	S	Branches, leaves	Dec	O,T	Anti-inflammatory	0.39	42
							Antidiarrheal		7
*Diospyros lotus* L/Amlook/B.Huss.80.UOP	Ebenaceae	W	Tr	Fruit	Dir	O	Cough	0.19	19
							Chest problems		6
*Elaeagnus angustifolia* L/Shangaly/B.Huss.81.UOP	Elaeagnaceae	W	Tr	Fruit, leaves	Dir, Dec	O	Antiseptic	0.07	7
							Colic pain		5
*Ephedra gerardiana* Wall. Ex. Stapf/Mawa/B.Huss.82.UOP	Ephedraceae	W	S	Whole plant	Dec	O	Antiasthmatic	0.24	25
							Stomachic		15
*Equisetum arvense* L /Bandokay/B.Huss.83.UOP	Equisetaceae	W	H	Aerial parts	Dir, Pow, Dec	O	Antiseptic	0.18	17
							Antidiarrheal		7
							Kidneys problems		3
*Eruca sativa* Mill/ Sharsham/B.Huss.84.UOP	Brassicaceae	C	H	Seed oil, leaves	Dir	O, T	Hair fall	0.14	14
							Nutritional		5
*Fagonia indica* L/Azghay/B.Huss.85.UOP	Zygophyllaceae	W	H	Aerial parts	Dec, Ash	O, T	Refrigerant	0.30	17
							Anti-inflammatory		7
							Blood purifier		31
*Ficus carica* L/Anzer/B.Huss.86.UOP	Moraceae	W	Tr	Fruit	Dir	O	Carminative	0.18	19
*Foeniculum vulgare* Miller/Khoglany/B.Huss.87.UOP	Apiaceae	W/C	H	Fruit, leaves	Dir	O	Colic pain	0.68	72
							Carminative		32
*Fragaria nubicola* (Hook.f.) Lindl. ex L/Manzakhka/B.Huss.88.UOP	Rosaceae	W	H	Fruit	Dir	O	Anemia	0.17	18
*Fumaria indica* (Hausskn.) Pugsle/Chamtara/chaptara/B.Huss.89.UOP	Fumariaceae	W	H	Aerial parts	Dec	O, T	Antiulcerogenic	0.41	13
							Antipyretic		4
							Blood purifier		44
							Emollient		7
							Itching		9
*Hordeum vulgare* L/Urbashy /B.Huss.90.UOP	Poaceae	C	H	Grains	J	O	Antidiabetic	0.10	11
*Juglans regia* L /Waghaz /B.Huss.91.UOP	Juglandaceae	C	Tr	Fruit, leaves	Dir	O	Brain tonic	0.49	53
							Dysentery		13
							Protect the teeth from decay		27
*Lepidium virginicum* L/Gharateraba/B.Huss.92.UOP	Brassicaceae	W	H	Leaves	Dir	O	Appetizer	0.05	5
*Mangifera indica* L/Aam/B.Huss.93.UOP	Anacardiaceae	C	Tr	Seeds	Pow	O	Antidiarrheal	0.17	13
							Dysentery		15
*Malva neglecta* Wallr /Tekalay/B.Huss.94.UOP	Malvaceae	W	H	Leaves, root	Dec	O	Dyspepsia	0.37	5
							Antiulcerogenic		37
							Carminative		17
*Marrubium vulgare* L/Dorshol/Butaka/B.Huss.95.UOP	Lamiaceae	W	H	Aerial parts	Dec	O, T	Antidiabetic	0.40	43
							Pimples treatment		12
*Melia azedarach* L/Daraka/B.Huss.96.UOP	Meliaceae	W	Tr	Leaves	Dec	O, T	Anti-dandruff	0.27	17
							Antidiabetic		28
							Hairs fall		14
*Mentha longifolia* (L.) Huds/Jangaliwilany/B.Huss.97.UOP	Lamiaceae	W	H	Aerial parts	Veg, Pow	O	Carminative	0.43	45
							Antidiarrheal		7
							Appetizer		12
*Mentha viridis* L/Podina/B.Huss.98.UOP	Lamiaceae	C	H	Aerial parts	Veg, Pow	O	Carminative	0.55	59
							Antidiarrheal		4
							Appetizer		9
*Momordica charantia* L/Karela/B.Huss.99.UOP	Cucurbitaceae	W	H	Fruit	Veg	O	Antidiabetic	0.62	67
*Morchella esculenta* Fr/Kerkachu/B.Huss.100.UOP--	Morchellaceae	W	H	Aerial parts	Ro	O	Nutritional	0.38	41
*Morus alba* L/Spin toot/B.Huss.101.UOP	Moraceae	W	Tr	Fruit	Dir	O	Laxative	0.26	28
*Morus nigra* L/Toor toot/B.Huss.102.UOP	Moraceae	W	Tr	Fruit	Dir	O	Laxative	0.41	26
							Cough		44
*Nannorrhops ritchiana* (Griff) Aitchison, J.L/Mazaray/B.Huss.103.UOP	Arecaceae	W	Tr	Fruit	Dir	O	Laxative	0.32	35
*Olea ferruginea* (Wall. Ex G. Don) Cif/Hamna/B.Huss.104.UOP	Oleaceae	W	Tr	Leaves	Dec	T	Joint pain	0.31	31
							Antidiabetic		18
*Onosma hispida* Wall. ex G. Don/Bezokhwnaiy/B.Huss.105.UOP	Boraginaceae	W	H	Aerial parts	J	T	Wound healing	0.12	13
							Antiseptic		5
*Oxalis corniculata* L/Bibishawtala/B.Huss.106.UOP	Oxalidaceae	W	H	Aerial parts	Dec	O	Kidneys problems	0.05	5
*Papaver somniferum* L/Dooda/B.Huss.107.UOP	Papaveraceae	C	H	Fruit	Inf	O	Cough	0.36	21
							Sedative		37
*Peganum harmala* L/Spinaly/B.Huss.108.UOP	Zygophyllaceae	W	H	Seeds	Dir	O	Obesity	0.18	19
*Pinus wallichiana* A.B. Jackson/Nekhter/B.Huss.109.UOP	Pinaceae	W	Tr	Resin, root	Res, Dec	O, T	Antiseptic	0.13	13
							Blood purifier		5
*Plantago lanceolata* L/Ghazaki/Palisepary/B.Huss.110.UOP	Plantaginaceae	W	H	Whole plant	Dec	O	Laxative	0.25	27
*Plantago major* L/Chanchapan/Ghuyezaba/B.Huss.111.UOP	Plantaginaceae	W	H	Whole plant	Dir, Dec	O, T	Wound healing	0.31	32
							Laxative		7
*Platanus orientalis* L/Chenoor/B.Huss.112.UOP	Platanaceae	W	Tr	Bark	Dec	T	Pimples treatment	0.12	13
*Polygonatum verticillatum* L/Nooryalam/B.Huss.113.UOP	Polygonaceae	W	H	Rhizome	Dec	O	Aphrodisiac	0.26	28
*Portulaca oleracea* L/Warkhuray/B.Huss.114.UOP	Aizoaceae	W	H	Aerial parts	Veg	O	Laxative	0.29	31
*Prunus jacquemontii* Hook/Arghanja/B.Huss.115.UOP	Rosaceae	W	S	Fruit	Dir	O	Hepatitis	0.17	18
*Punica granatum* L/Wangar/B.Huss.116.UOP	Punicaceae	W	S	Fruit	Dir, Pow	O	Cough	0.29	12
							Antidiarrheal		29
							Antiulcerogenic		9
							Dysentery		17
							Eye disorders		3
*Quercus baloot* Griff/Sayreye/B.Huss.117.UOP	Fagaceae	W	S	Fruit	Dec	O	Antidiabetic	0.19	18
							Antiulcerogenic		8
*Raphanus sativus* L/Moli/B.Huss.118.UOP	Brassicaceae	C	H	Root	Dir	O	Blood pressure	0.22	8
							Hepatitis		23
*Rheum speciforme* Royle/Pakhey/B.Huss.119.UOP	Polygonaceae	W	H	Petioles	Dir	O	Cardio tonic	0.12	13
*Robinia pseudo-acacia* L/Chanbele/B.Huss.120.UOP	Fabaceae	W	Tr	Inflorescence	Dec	O	Laxative	0.16	15
							Nutritional		10
*Rosa moschata* J. Herm/JangliGulab/B.Huss.121.UOP	Rosaceae	W	S	Petals	Dec	O	Laxative	0.30	28
							Expectorant		16
*Rosa webbiana* Wall ex. Royle/Jangaligulab/B.Huss.122.UOP	Rosaceae	W	S	Fruit, seeds	Dir	O	Carminative	0.21	23
*Rubus fruitcosus* L/GharyManzakhka/B.Huss.123.UOP	Rosaceae	W	S	Fruit	Dir	O	Anemia	0.26	28
*Rumex dentatus* L/Zamda/B.Huss.124.UOP	Polygonaceae	W	H	Root	J	O	Antiseptic	0.17	8
							Antiulcerogenic		17
*Rununculus muricatus* L/Zergulak/B.Huss.125.UOP	Ranunculaceae	W	H	Leaves	J	O	Analgesic	0.04	4
*Salvia reflexa* Hormn/Sugar boti/B.Huss.126.UOP	Lamiaceae	W	H	Aerial parts	Dec	O	Antidiabetic	0.25	27
*Sambucus nigra* L/Lantus/B.Huss.127.UOP	Sambucaceae	W	S	Fruit	Inf	O	Flu	0.16	17
*Seriphidium kurramensis* (Qazilb.) Y. R. Sling/Tarkha/B.Huss.128.UOP	Asteraceae	Q	US	Aerial parts	Dec	O	Antipyretic	0.51	15
							Anthelmintic		30
							Antimalarial		54
*Solanum nigrum* L/Bartang/Kharsobay/B.Huss.129.UOP	Solanaceae	W	H	Fruit, leaves	Inf	O	Antidiarrheal	0.10	3
							Antidiuretic		6
							Hepatitis		7
*Solanum surattense* Burm. f/Marghony/B.Huss.130.UOP	Solanaceae	W	H	Fruit	Dec	O	Cough	0.13	14
*Sonchus asper* (L.) Hill/Tareza/B.Huss.131.UOP	Asteraceae	W	H	Leaves, root	Inf	O	Hepatitis	0.14	15
*Tanacetum artemisioides* L/Zawil/B.Huss.132.UOP	Asteraceae	W	H	Aerial parts	Inf	O	Antidiabetic	0.20	21
							Antiseptic		4
							Cough		7
							Hepatitis		9
*Taraxicum officinale* L/Chechopaska/B.Huss.133.UOP	Asteraceae	W	H	Aerial parts	Dec	O	Tonic	0.34	15
							Hepatitis		36
*Teucrium stocksianum B* Boiss/Harboty/Gulbahar/B.Huss.134.UOP	Lamiaceae	W	H	Aerial parts	Inf	O	Anthelmintic	0.14	13
							Antidiabetic		9
							Antidiuretic		2
							Antipyretic		6
*Thymus linearis* L/Paney/Mawory/B.Huss.135.UOP	Lamiaceae	W	H	Aerial parts	Inf	O	Cough	0.56	47
							Appetizer		15
							Carminative		53
*Tulipa clusiana* DC/Spergha/B.Huss.136.UOP	Liliaceae	W	H	Rhizome	Pow	O	Anthelmintic	0.19	21
*Urtica dioica* L/Sezonky/B.Huss.137.UOP	Urticaceae	W	H	Aerial parts	Dec	O	Anti-inflammatory	0.19	17
							Joint pain		15
*Valeriana jatamansi* Jones/Mehkek/B.Huss.138.UOP	Valerianaceae	W	H	Root	Pow	O	Aphrodisiac	0.22	24
*Verbascum thapsus* L/Kharghogy/B.Huss.139.UOP	Scrophulariaceae	W	H	Leaves	J	O,T	Antiseptic	0.14	15
							Ear problems		3
*Vincetoxicum cardiostephanum* (Rech.f) Rech.f/Kamyaboti/B.Huss.140.UOP	Asclepiadaceae	W	H	Whole plant	Dec	O	Chest problems	0.18	19
*Viola canescens* Wall ex Roxb/Benefsha/balamsha/B.Huss.141.UOP	Violaceae	W	H	Leaves, rhizome	Dec	O	Chest problems	0.26	14
							Cough		25
*Withania coagulans* (Stocks) Dunal in DC/hapyanaga/hafyanga/Shapynga/B.Huss.142.UOP	Solanaceae	W	US	Fruit	Pow	O	Stomach pain	0.96	103
							Antidiabetic		9
							Antiulcerogenic		14
							Constipation		50
*Xanthium strumarium* L/Azghy/B.Huss.143.UOP	Asteraceae	W	H	Fruit	J	T	Skin problems	0.09	10
*Zea mays* L/Jawar/B.Huss.144.UOP	Poaceae	C	H	Grains	Dir	O	Obesity	0.07	8
*Zingeber officinale* L/Adrek/B.Huss.145.UOP	Zingiberaceae	C	H	Rhizome	Dec	O	Cough	0.43	46
*Ziziphora tenuior* L/Sahrawaleny/B.Huss.146.UOP	Lamiaceae	W	H	Leaves	Pow	O	Appetizer	0.18	16
							Carminative		12

*Abbreviations*: *C* cultivated, *Dec* decoction, *Dir* direct, *FC* frequency of citation, *H* herb, *J* juice, *O* orally, *Pow* powder, *Res* resin, *S* shrub, *RFC* relative frequency citation, *Ro* roast *ROA* route of administration, *T* topically, *T* tree, *US* undershrub, *UV* use value, *Veg* vegetable, *W* wild

### Plant parts used in preparation of remedies

**Fig. 3:**
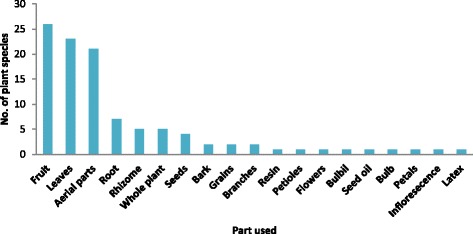
Plant parts used in the formulation of remedies

The plant parts used in the preparation of remedies were root, rhizome, bulbils, stem, branches, leaves, flowers, fruits, seeds, bark, resin, and latex. The relative use of these plant parts is shown in (Fig. 3). Fruits were frequently used plant part (26 species), followed by leaves (23 species) and remaining parts (21 species).

### Preparation and mode of administration of remedies

**Fig. 4:**
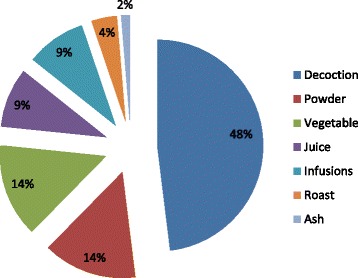
Different modes of drug formulation

**Fig. 5:**
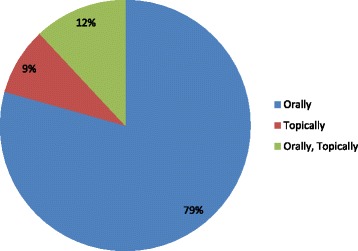
Route of administration of drugs

The collection of data for the preparation of remedies from medicinal plants is extremely important. Such information is essential for identification of active ingredients and intake of relevant amount of drug. The present research observed seven methods for preparing recipes. It included decoction, powder, juice, infusions, roast, and ash methods (Fig. 4). The 37 species (40%) were most frequently used for the preparation of remedies. A plant part is boiled while infusion is obtained by soaking plant material in cold or hot water overnight. Eleven species (14%) are in powdered form, 11species (14%) in vegetable form, 7 species (9%) in juice form, 7 species (9%) in infusions form, 3 species (4%) in roasted form, and 1 species (2%) in ash form were used. Twenty-seven plant parts were used directly. It included wild fruits that were consumed for their nutritional and medicinal purpose. The most frequently used mode of administration of remedies was oral intake practice of 74 species (79%) followed by both orally and topically practice of 11 species (12%) and topically of 8 species (9%) (Fig. 5).

### Medicinal plants use categories

The inhabitants used medicinal plants in the treatment of 53 health disorders. The important disorders were cancer, diabetic, diarrhea, dysentery, hepatitis, malaria, and ulcer (Table. 3). These disorders were classified into 17 categories. Among the ailments, most plants were used for the treatment of digestive problems mainly as carminative (12 species), diarrhea (11 species), laxative (11 species), ulcer (7 species), appetizer (5 species), colic pain (4 species), and anthelmintic (4 species). Such higher use of plants for the treatment of digestive problems had been reported in ethnobotanical studies conducted in another tribal area of Pakistan [21]. The other categories (18 species) were used to treat respiratory disorders, followed by endocrine disorders (16 species); antiseptic and anti-inflammatory (15 species); circulatory system disorders (15 species); integumentary problems (15 species); antipyretic, refrigerant, and analgesic (9 species); and hepatic disorders (9 species). However, among the ailments, the highest number of plants were used in the treatment of diabetes (16 species), followed by antiseptic (11 species), cough (10 species), hepatitis (9 species), and ulcer (7 species). Among the remaining species, the informants reported three and two species used against malaria and cancer, respectively (Table 3).

**Table 3:** Medicinal plants and their use categories

Medicinal use categories	Number of plants used for each ailment
Digestive disorders	Carminative (12), diarrhea (11), laxative (11), ulcer (7), appetizer (5), colic pain (4), anthelmintic (4), stomach pain (3), dysentery (3), stomachic (2), constipation (1), dyspepsia (1)
Respiratory disorders	Cough (10), chest problems (4), asthma (1), expectorant (1), dyspnea (1), flu (1)
Endocrine disorders	Antidiabetic (16)
Antiseptic and anti-inflammatory	Antiseptic (11), anti-inflammatory (4)
Circulatory disorders	Blood purifier (5), blood pressure (4), malaria (3), anemia (2), cardio tonic (1)
Integumentary disorders	Wound healing (5), skin problems (2), pimple treatment (2), hair fall (2), anti-dandruff (1), emollient (1), itching (1)
Antipyretic, refrigerant, analgesic	Antipyretic (5), refrigerant (3), analgesic (1)
Hepatic disorders	Hepatitis (9)
Nutritional problems and tonic	Nutritional (3), obesity (2), tonic (2), hypolipidemic (1)
Urologic disorders	Antidiuretic (2), kidney problems (2), urologic problems (1)
Nervous disorders	Sedative (2), brain tonic (1)
Skeletal disorders	Joint pain (3)
Cancer	Anticancer (2)
Ophthalmic disorders	Eye disorders (2)
Sexual disorders	Aphrodisiac (2)
Auditory disorders	Ear problems (1)
Dental disorders	Tooth decay (1)

### Quantitative appraisal of ethnomedicinal use

**Fig. 6:**
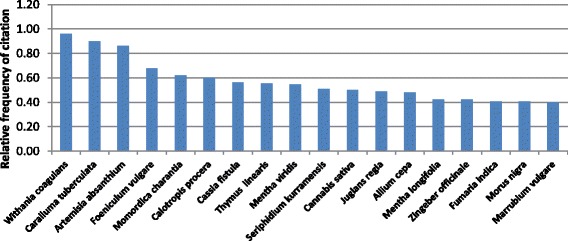
Medicinal plants with highest relative frequency citation

**Fig. 7:**
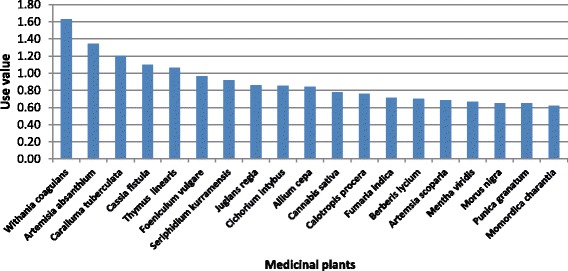
Medicinal plants with highest relative importance

Based on the quantitative indices, the analyzed data showed that few plants were cited by the majority of the informants for their medicinal value. Seventeen plant species with the highest citation frequency are shown in (Fig. 6). The highest citation frequency was calculated for *Withania coagulans* (0.96), followed by *Caralluma tuberculata* (0.90), and *Artemisia absanthium* (0.86). The high values of these species indicated that most of the informants were familiar with their medicinal value. However, the familiarity of these three plants could be linked to their collection for economic purposes [22]. *Withania coagulans* (1.63), *Artemisia absinthium* (1.34), *Caralluma tuberculata* (1.20), *Cassia fistula* (1.10), and *Thymus linearis* (1.06) were reported having the highest used values for medicinal purposes (Fig. 7). All these species were used for the cure of three or more diseases. The powdered fruit of *Withania coagulans* is used for the cure of stomach pain, constipation, diabetes, and ulcer. The next highest use value was calculated for *Artemisia absinthium* with five medical indications as diabetes, malaria, fever, blood pressure, and urologic problems. Among the remaining three plants, *Caralluma tuberculata* is used for diabetes, cancer, and stomachic problems, and as blood purifier; *Cassia fistula* for colic pain and stomach pain and as a carminative agent; and *Thymus linearis* for cough and as carminative and appetizer. Lowest use value was calculated for *Rununculus muricatus* (0.04) with next three species having same lowest use value: *Abies pindrow* (0.05), *Lepidium virginicum* (0.05), and *Oxalis corniculata* (0.05). Highest family use value was calculated for Juglandaceae (0.86), followed by Cannabaceae (0.78), Apiaceae (0.75), Asclepiadaceae (0.71), Fumariaceae (0.71), Berberidaceae (0.70), Fabaceae (0.67), Punicaceae (0.65), Solanaceae (0.64), and Asteraceae (0.61). This is the first study that presents a quantitative value of medicinal plants used in the investigated area.

### Conservation status of the medicinal flora

Plant preservation means the study of plant declination, their causes, and techniques to protect rare and scarce plants. Plant conservation is a fairly new field that emphasizes the conservation of biodiversity and whole ecosystems as opposed to the conservation of individual species [23]. The ex situ conservation must be encouraged for the protection of medicinal plants [24]. In the present case, the area under study is under tremendous anthropogenic pressure as well. Therefore, ex situ conservation of endangered species is recommended. The woody plants, cut down for miscellaneous purposes, are facing conservational problems. Sayer et al. [25] reported that large investments are being made in the establishment of tree plantation on degraded area in Asia [25]. Alam and Ali stressed that proper conservation studies are almost negligible in Pakistan [26]. Same is the case with the study area as no project has been initiated for the conservation of forest or vegetation so far. Anthropogenic activities, small size population, distribution in limited area, and specificity of habitat were observed as the chief threats to endangered species.

According to IUCN Red List Criteria (2001) [18] conservation status of 80 wild medicinal species have been assessed based on availability, collection status, growth status, and their parts used. The remaining 12 medicinal plants were cultivated species. Of these, 7 (8.7%) species are endangered, 34 (42.5%) species are vulnerable, 29 (36.2%) species are rare, 9 (11.2%) species are infrequent, and only 1 (1.3%) species is dominant. The endangered species were *Caralluma tuberculata*, *Morchella esculenta*, *Rheum speciforme*, *Tanacetum artemisioides*, *Vincetoxicum cardiostephanum*, *Withania coagulans*, and *Polygonatum verticillatum.*

## Discussion

Traditional medicines are a vital and often underestimated part of health care. Nowadays, it is practiced in almost every country of the world. Its demand is currently increasing rapidly in the form of alternative medicine [20]. Ethnomedicinal plants have been widely applied in traditional medicine systems to treat various ailments. About 80% of the populations in developing countries rely on medicinal plants to treat diseases, maintaining and improving the lives of their generation [19]. Traditional knowledge has a long historical cultural heritage and rich natural resources that has accumulated in the indigenous communities through oral and discipleship practices [8]. Traditional indigenous knowledge is important in the formulation of herbal remedies and isolates bioactive constituents which are a precursor for semisynthetic drugs. It is the most successful criterion for the development of novelties in drugs [11]. A total of 92 medicinal species including 91 vascular plant species belonging to 50 families and 1 mushroom *Morchella* of Ascomycetes of family Morchellaceae were reported (Table 2)*.* The current study reveals that the family Asteraceae represents eight species followed by seven species of Lamiaceae and Rosaceae each which showed a higher number of medicinal plants. Three species were contributed by each of Moraceae, Asclepiadaceae, Polygonaceae, Brassicaceae, Solanaceae, Cucurbitaceae, and Amaryllidaceae. While the remaining eight families, namely, Poaceae, Pinaceae, Zingiberaceae, Chenopodiaceae, Plantaginaceae, Apiaceae, Fabaceae, and Zygophylaceae, contributed two species each. Asteraceae, Lamiaceae, and Rosaceae were also reported with a high number of plants used for medicinal purposes. Indigenous use of medicinal plants in the communities residing in Koh-e-Safid Range of Pakistan is evident. Traditional health healers are important to fulfill the basic health needs of the economically poor people of the area. The high dependency on traditional healers is due to limited and inaccessible health facilities. Most people either take recipes from local healers or select wild medicinal plants prescribed by them. Some elders also knew how to preserve medicinal plant parts for future use. Traditional knowledge of medicinal plants is declining in the area due to lack of interest in the young generation to acquire this traditional treasure. Furthermore, most traditional health healers and knowledgeable elders hesitate to disseminate their recipes. Therefore, traditional knowledge in the area is diminishing as aged persons are passing away. Vernacular names of plants are the roots of ethnomedicinal diversity knowledge [27]. They can clear the ambiguity in the identification of medicinal plants within an area. It also helps in the preservation of indigenous knowledge of medicinal plants. The medicinal plants were mostly reported with one specific vernacular name in the investigated area. While *Rosa moschata* and *Rosa webbiana* were known by same single vernacular name as Jangle Gulab. Few species were known by two vernacular names: *Curcuma longa* as Korkaman or Hildi, *Ficus carica* as Togh or Anzer, *Fumaria indica* as Chamtara or Chaptara, *Marrubium vulgare* as Dorshol or Butaka, *Solanum nigrum* as Bartang or Kharsobay, *Teucrium stocksianum* as Harboty or Gulbahar, and *Thymus linearis* as Paney or Mawory. The informants also mentioned different vernacular names for species even belonging to single genus; *Plantago lanceolata* as Chamchapan or Ghuyezaba and *Plantago major* as Ghazaki or Palisepary. Majority of the species commonly had a single name. However, local dialects varied in few species, i. e., *Withania coagulans* was known by three names: Hapyanaga, Hafyanga, and Shapynga, *Caralluma tuberculata* as Pamenny or Pawanky, *Foeniculum vulgare* as Koglany or Khoglany, and *Viola canescens* was called as Banafsha or Balamsha. The species with high use value need conservation for maintaining biodiversity in the study area. However, in the present case, no project or programs for the conservation of forest or vegetation are operating. Grazing and unsustainable medicinal uses were observed as the chief hazard to highly medicinal plant species. The higher use of herbs can be attributed to their abundance, diversity, and therapeutic potentials as antidiabetic, antimalarial, antipyretic, antiulcerogenic, antipyretic, blood purifier, and emollient and for blood pressure, hepatitis, stomach pain, and itching. *Aloe vera*, cultivated for ornamental purpose, is used as wound healing agent. Among the plant parts, the higher use of fruit may relate to its nutritional value. The aerial parts of the herbaceous plants were mostly collected in abundance and frequently used for medicinal purposes. In many recipes, more than one part was used. The utilization of roots, rhizomes, and the whole plant is the main threat in the regeneration of the medicinal plants [28]. In the current study, decoction was found to be the main method of remedy preparation as reported in the ethnopharmacological studies from other parts [29–31]. Fortunately, we collected important information like preparation of remedies and their mode of administration for all the reported plants. However, the therapeutic potential of few plants are connected to their utilization method. A roasted bulb of *Allium cepa* is wrapped on the spine-containing wound to release the spine. The leaf of *Aloe vera* containing viscous juice is scratched and wrapped on a wound. The latex of *Calotropis procera* is first mixed with flour and then topically applied on the skin for wound healing. Infusion of *Cassia fistula* fruit’s inner septa is prepared for stomach pain and carminative and colic pain in children. The fruit of *Citrullus colocynthis* boiled in water is orally taken for the treatment of diabetes. Grains of *Hordeum vulgare* are kept in water for a day, and its extraction is taken for the treatment of diabetes. The decoction of *Seriphidium kurramensis* shoots are used as anti-anthelmintic and antimalaria. The leaves of *Juglans regia* are locally used for cleaning the teeth and to prevent them from decaying. Furthermore, its fruit is used as brain tonic, and its roasted form is useful in the treatment of dysentery. The roots of *Pinus wallichiana* are cut into small pieces and put into the pot. The cut pieces are boiled, and the extracted liquid is poured into the container. One drop of the extracted liquid is mixed with one glass of milk and taken orally once a day as blood purifier. An infusion of *Thymus linearis* aerial parts is prepared like hot tea and is drunk for cough and as appetizer and carminative. A decoction of *Zingiber officinale* rhizome is drunk at night time for relief of cough. Medicinal plants are still practiced in tribal and rural areas as they are considered as main therapeutic agents in maintaining better health. Such practices have been described in the ethnobotanical studies conducted across Pakistan. The current study reveals several plant species with more than one medical use including *Artemisia absanthium*, *Cichorium intybus*, *Fumaria indica*, *Punica granatum*, *Tanacetum artemisioides*, *Teucrium stocksianum*, and *Withania coagulans*. Their medicinal importance can be validated from indigenous studies conducted in various parts of the country. *Amaranthus viridis* leaf extract is an emollient and is used for curing cough and asthma as well [32]. *Artemisia absanthium* is used for the treatment of malaria and diabetes [33–36]. *Cichorium intybus* is used against diabetes, malaria, and gastric ulcer, and it is also used as digestive and laxative agent [28, 37–41]. Leaves of *Cannabis sativa* are used as bandage for wound healing; powdered leaves as anodyne, sedative, tonic, and narcotic; and juice added with milk and nuts as a cold drink [42]. Whole plant of *Fumaria indica* [36] and *Tanacetum artemisioides* [43] is used for treating constipation and diabetes, respectively. Dried rind powder and fruit extract of *Punica granatum* are taken orally for the treatment of anemia, diarrhea, dysentery, and diabetes [44–47]. A decoction of aerial parts of *Teucrium stocksianum* is used for curing diabetes [29, 48]. *Withania coagulans* is known worldwide [38, 49] as a medicinal plant, whose fruit decoction is best remedy for skin diseases and diabetes. Its seeds are used against digestive problems, gastritis, diabetes, and constipation [21, 28, 50]. Our results are in line with the traditional uses of plants in the neighboring counties [8]. For example, *Fumaria indica* is used as blood purifier, and *Hordeum vulgare* grains decoction for diabetes; *Juglans regia* bark for toothaches and scouring teeth; *Mangifera indica* seed decoction for diarrhea; *Solanum nigrum* extract for jaundice; and *Solanum surattense* fruit decoction for cough have been documented in the study (40)*.* Such agreements strengthen our results and provide good opportunity to evaluate therapeutic potential of the reported plants. Three plants species *Adiantum capillus-veneris*, *Malva parviflora*, and *Peganum harmala* have been documented for their medicinal use in the ethnobotanical study [51]. According to this, the decoction of the aerial parts of *Adiantum capillus-veneris* is used for the treatment of asthma and dyspnea. *Malva parviflora* root and flower are used for stomach ulcers. *Peganum harmala* fruit powder and decoction are used for toothache, gynecological infections, and menstruation. The dried leaves of *Artemisia absanthium* is used to cure stomach pain and intestinal worm while an inflorescence paste prepared from its fresh leaves is used as wound healing agent and antidiabetic [52, 53]. The bulb of *Allium sativum* is used in rheumatism while its seed vessel mixed with hot milk is useful for the prevention of tuberculosis and high blood pressure. The fruit bark of *Punica granatum* is used in herbal mixture for intestinal problems [54]. *Avena sativa* decoction is used for skin diseases including eczema, wounds, irritation, inflammation, erythema, burns, itching, and sunburn [55]. *Foeniculum vulgare* and *Lepidium sativum* are used for the treatment of diabetes and renal diseases [53]. *Verbascum thapsus* leaves and flowers can be used to reduce mucous formation and stimulate the coughing up of phlegm. Externally, it is used as a good emollient and wound healer. Leaves of *Thymus linearis* are effective against whooping cough, asthma, and round worms and are an antiseptic agent [21]. *Berberis lycium* wood decoction with sugar is the best treatment for jaundice. *Chenopodium album* has anthelmintic, diuretic, and laxative properties, and its root decoction is effective against jaundice. The whole plant decoction of *Fumaria indica* is used for blood purification. Dried leaves and flowers of *Mentha longifolia* are used as a remedy for jaundice, fever, asthma, and high blood pressure [36]. *Morus alba* fruit is used to treat constipation and cough [42]. *Oxalis corniculata* roots are anthelmintic, and powder of *Chenopodium album* is used for headache and seminal weakness [47]. Boiled leaves of *Cichorium intybus* are used for stomachic pain and laxative while boiled leaves of *Plantago major* are used against gastralgia [56]. *Viola canescens* flower is used as a purgative [32]. The above ethnomedicinal information confirms the therapeutic importance of the reported plants. The reported plant species show biological activities which suggest their therapeutic uses. The aqueous extract of *Allium sativum* has been studied for its lipid lowering ability and was found to be effective at the amount of 200 mg/kg of body weight. It also has significant antioxidant effect and normalizes the activities of superoxide dismutase, catalase, glutathione peroxidase, and glutathione reductase in the liver [57]. An extract of *Artemisia absanthium* antinociception in mice has been found and was linked to cholinergic, serotonergic, dopaminergic, and opioidergic system [58]. The ethanolic extract of *Artemisia absanthium* at a dose of 500 and 1000 mg/kg body weight has reduced blood glucose to significant level [59]. The hepatoprotective activity of crude extract of aerial parts of *Artemisia scoparia* was investigated against experimentally produced hepatic damage through carbon tetrachloride. The experimental data showed that crude extract of *Artemisia scoparia* is hepatoprotective [60]. Ethanolic and aqueous extracts from Asparagus exhibited strong hypolipidemic and hepatoprotective action when administered at a daily dose of 200 mg/kg for 8 weeks in hyperlipidemic mice [61, 62]. The extract of *Calotropis procera* was evaluated for the antiulcerogenic activity by using different in vivo ulcer in pyloric-ligated rats, and significant protection was observed in histamine-induced duodenal ulcers in guinea pigs [63]. Cannabidiol of *Cannabis sativa* was found as anxiolytic, antipsychotic, and schizophrenic agent [64]. *Caralluma tuberculata* methanolic extract of aerial parts (500 mg/kg) in fasting blood glucose level in hyperglycemic condition decreased up to 54% at fourth week with concomitant increase in plasma insulin by 206.8% [65]. The aqueous and methanol crude extract of *Celtis australis*, traditionally used in Indian system of medicine, was screened for its antibacterial activity [66]. *Cichorium intybus* L. whole plant 80% ethanolic extract a percent change in serum glucose has been observed after 30 min in rats administrated with vehicle, 125, 250, and 500 mg notified as 52.1, 25.2, 39, and 30.9%, respectively [67]. *Citrullus colocynthis* fruit, pulp, leaves, and root have significantly decreased blood glucose level and restored beta cells [30, 68–70]. The two new aromatic esters horizontoates A and B and one new sphingolipid C were isolated from *Cotoneaster horizontalis*. The compounds A and B showed significant inhibitory effects on acetylcholinesterase and butylcholinesterase in a dose-dependent manner [71]. The alkaloids found in *Datura stramonium* are organic esters used clinically as anticholinergic agents [72]. The methanolic extract of *Momordica charantia* fruits on gastric and duodenal ulcers was evaluated in pylorus-ligated rats; the extract showed significant decrease in ulcer index [73]. Antifungal activity of *Nannorrhops ritchiana* was investigated against fungal strains *Aspergillus flavus*, *Trichophyton longifusis*, *Trichophyton mentagrophytes*, *Aspergillus flavus*, and *Microsporum canis* were found susceptible to the extracts with percentage inhibition of 70–80% [74]. The inhibitory effects of *Olea ferruginea* crude leaf extract on bacterial and fungal pathogens have been evaluated [75]. The aqueous extract of *Plantago lanceolata* showed that higher doses provide an overall better protection against gastro-duodenal ulcers [76]. The oral and intraperitoneal management of extracts reduced the gastric acidity in pylorus-ligated mice [77]. The antiulcer effect of *Solanum nigrum* fruit extract on cold restraint stress, indomethacin, pyloric ligation, and ethanol-induced gastric ulcer models and ulcer healing activity on acetic acid-induced ulcer model in rats [78, 79]. The antifungal activity (17.62 mm) of *Viola canescens* acetone extract 1000 mg/ml against *Fusarium oxysporum* has been observed [80]. Leaf methanolic extract of *Xanthium strumarium* has inhibited eight pathogenic bacteria at a concentration of 50 and 100 mg/ml [81]. Aqueous extract of the fruits of *Withania coagulans* in streptozotocin-induced rats at dose of 1 g/kg for 7 days has shown significant decrease (*p* < 0.01) in the blood glucose level (52%), triglyceride, total cholesterol, and low density lipoprotein and very significant increase (*p* < 0.01) in high density lipoprotein level [31]. This shows that further investigation on the reported ethnomedicinal plants can lead to the discovery of novel agents with therapeutic properties.

In the current study, conservation status of 80 medicinal species was reported which was growing wild in the area. The information was collected and recorded for different conservation attributes by following International Union for Conservation and Nature (2001) [18]. It was reported that seven species (8.7%) were endangered due to the much collection, anthropogenic activities, adverse climatic conditions, small size population and distribution in limited area, specificity of habitat, and over grazing in the research area. However, the below-mentioned species were found to be endangered: *Caralluma tuberculata*, *Morchella esculenta*, *Rheum speciforme*, *Tanacetum artemisioides*, *Vincetoxicum cardiostephanum*, *Withania coagulans*, and *Polygonatum verticillatum*. Unsustainable use and lack of suitable habitat have affected their regeneration and pushed them to endangered category. Traditional knowledge can also contribute to conservation and sustainable use of biological diversity [19, 20].

### Novelty and future prospects

Ethnomedicinal literature research indicated that five plant species, *Abies pindrow*, *Artemisia scoparia*, *Nannorrhops ritchiana*, *Salvia reflexa*, and *Vincetoxicum cardiostephanum*, have not been reported previously for their medicinal importance from this area. The newly documented uses of these plants were *Abies pindrow* and *Salvia reflexa* (antidiabetic), *Artemisia scoparia* (anticancer), *Nannorrhops ritchiana* (laxative), and *Vincetoxicum cardiostephanum* (chest problems). *Adiantum capillus-veneris* is reported for the first time for its use in the treatment of skin problems. These plant species can be further screened for therapeutic agents and their pharmacological activities in search of novel drugs. The study also highlights 16 species of antidiabetic plants *Caralluma tuberculata*, *Momordica charantia*, *Marrubium vulgare*, *Artemisia scoparia*, *Melia azedarach*, *Salvia reflexa*, *Citrullus colocynthis*, *Tanacetum artemisioides*, *Quercus baloot*, *Olea ferruginea*, *Cichorium intybus*, *Artemisia absinthium*, *Hordeum vulgare*, *Teucrium stocksianum*, *Withania coagulans*, and *Abies pindrow*. Except sole paper from District Attack, Pakistan [28], such a high number of antidiabetic plants have not been reported previously from any part of Pakistan in the ethnobotanical studies.

## Conclusions

Traditional knowledge about medicinal plants and preparation of plant-based remedies is still common in tribal area of Koh-e-Safaid Range. People due to closeness to medicinal plants and inaccessible health facilities still rely on indigenous traditional knowledge of plants. The role of traditional healers in the area is observable in primary health care. The locals used medicinal plants in treatment of important disorders such as cancer, diabetes, hepatitis, malaria, and ulcer. The analyzed data may provide opportunities for extraction of new bioactive constituents and to develop herbal remedies. The study also confirmed that the communities residing in the area have not struggled for conservation of this traditional treasure of indigenous knowledge and medicinal plants. Medicinal plant diversity in the remote and backward area of Koh-e-Safaid Range has great role in maintaining better health conditions of local communities. Therefore, conservation strategies should be adopted for the protection of medicinal plants and traditional knowledge in the study area to sustain them in the future.

## Additional files

Additional file 1: Field data of the research project Quantitative study of medicinal plants used by the communities residing in Koh-e-Safaid Range northern Pakistani-Afghan border. (XLSX 167 kb)

Additional file 2: Annexures. (DOCX 27 kb)

## Abbreviations

FUV: Family use value
IUCN: International Union for Conservation of Nature
Ni: The number of informants
Ns: Represent the number of species reported for the family
UV: Use value
UVi: The number of citations for a species across all informants
UVs: Represent sum of use values of species falling within family
