# Supplementary material for: Quantitative study of medicinal plants used by the communities residing in Koh-e-Safaid Range, northern Pakistani-Afghan borders
Source: J Ethnobiol Ethnomed. 2018 Apr 25;14:30. doi: 10.1186/s13002-018-0229-4 (PMC5922303; doi:10.1186/s13002-018-0229-4)
Supplement: Supplementary file 2 — Annexures. (DOCX 27 kb) [file 13002_2018_229_MOESM2_ESM.docx]

**Questionnaire for medicinal plant collectors**

1. Name of Person _____________________________
2. Age _____________________________
3. Education ______________________________
4. Status as a plant collector
   1. Permanent (year spent) ____________________________
   2. Occasional ______________________________
5. Reason for collection
   1. For domestic purpose______________________________
   2. For commercial purpose (sale) ______________________________

| Plant Name | Local Name | Habit | Locality | Sub Locality | Region | Uses | Recipes |
| --- | --- | --- | --- | --- | --- | --- | --- |
|  |  |  |  |  |  |  |  |

1. Name and uses of plant species which you collect.
2. Name of trips per week for plant collection ___________________________.
3. What is the best possible season for plant collection ____________________.
4. Quantity of plant collection (kg/week) ______________________________.
5. Do you sell these medicinal plants? Y/N
   1. if yes then where (market place)
   2. Sale rate Rs/kg______________________________
6. How many people involved in this profession? ______________________.
7. Competition for plant collection in the last twenty years (trend of collectors) increase/decrease.

a. if increase or decrease then given reasons. ________________________

1. Present competition for plant collection, increase/decrease

a. If increase or decrease then give reasons__________________________

15. Have you any problem in the collection of these plants? Y/N

a. If yes then what kind of problems_______________________.

i. Far away

ii. Threats from big game animals.

iii. Problems of thieves.

iv. Others

16. Do you think that the availability of these plants have been increased/decreased?

- 1. If decreased then what are the reasons? __________________________

1. Overgrazing

ii. Grass cutting

iii. Fuel wood collection

1. Agricultural land expansion
2. Others

17. Have you tried to cultivate any plant (especially Medicinal)? Y/N

- 1. . If Yes then what type__________________________.

18. Will you cooperate if the organization initiates some conservation activities in your area?

Y/N____________________________________________________

19. Any suggestion for the sustainable use of natural resources.

__________________________________________________________________

**Questionnaire for medicinal plants venders**

1. Name ________________________________________

2. Village ________________________________________

3. Market Place ________________________________________

4. Age ________________________________________

5. Education ________________________________________

6. How many years have you spent in this profession/when you started this business?

c. Permanent________________________________________

d. Temporary________________________________________

e. Ambulatory________________________________________

7. From where you get the medicinal plants. ___________________________________.

a. By himself________________________________________

b. From other body. _______________________________________

8. If from other body then.

a. From which village________________________________________ .

b. Collector address________________________________________.

c. Collection on weekly/monthly basis_________________________________.

d. Purchase Price Rs/kg________________________________________.

e. Sale rate Rs/Kg________________________________________.

f. Quantity sold per annum in kg __________________________________.

9. What is the best favorable season for the collection of medicinal plants? ___________.

10. Name and uses of plants (medicinal) which you collect.

| Vernacular Name | Botanical Name | Uses |
| --- | --- | --- |
|  |  |  |
|  |  |  |
|  |  |  |
|  |  |  |
|  |  |  |
|  |  |  |
|  |  |  |
|  |  |  |

11. How many venders are there in this market, their name and address?

____________________________________________________________________________________________________________________________________

12. Have you any link with Afghanistan for the import of medicinal plants Y/N?

a. if yes then from which province and what types.

| Province | Type of medicinal plant Fresh form/dry form | Purchase rate aRs/kg/month | Sale rate Rs/kg/month |
| --- | --- | --- | --- |
|  |  |  |  |
|  |  |  |  |
|  |  |  |  |
|  |  |  |  |

13. Do you process these medicinal plants Y/N? _______________________________.

14. Do you think that there is any threat to medicinal plants Y/N?

c. If yes then what kind of threats.

________________________________________________________________________________________________________________________________________________

15. People’s trend to towards using your traditional medicines increase/decrease_______.

a. If increase or decrease then give reasons.

________________________________________________________________________________________________________________________________________________

16. How many patients visits you daily (their number) ___________________________.

17. How many medicines you sell daily (its price) _______________________________.

18. Do you think that your business remained same, increase, decrease as compared to past twenty years? ________________________________________.

19. If decrease, increase or same then why.

a. Supply and demand has been decrease.

b. People’s trend towards using allopathic drugs has decreased.

c. Medicinal plants are going to be depleted day by day due to over harvesting for multiple purposes like grazing grass cutting and fuel wood collection.
